# Bilateral Sclerosing Stromal Ovarian Tumor in an Adolescent

**DOI:** 10.1155/2015/271394

**Published:** 2015-05-06

**Authors:** Anjani Naidu, Betty Chung, Mitchell Simon, Ian Marshall

**Affiliations:** ^1^Department of Radiology, Rutgers Robert Wood Johnson Medical School, 1 Robert Wood Johnson Place, MEB 404, New Brunswick, NJ 08901, USA; ^2^Department of Pathology, Rutgers Robert Wood Johnson Medical School, 1 Robert Wood Johnson Place, New Brunswick, NJ 08901, USA; ^3^Division of Pediatric Endocrinology, Rutgers Robert Wood Johnson Medical School, Child Health Institute of New Jersey, 89 French Street, New Brunswick, NJ 08901, USA

## Abstract

Sclerosing stromal tumor of the ovary is a rare, benign, sex cord stromal tumor occurring predominantly in younger women in the 2nd and 3rd decades of life. It typically presents unilaterally with only 2 previously reported cases of bilateral presentation. Common clinical presentations include pelvic or abdominal pain, a mass, or menstrual changes. Although occasionally presenting with hormonal manifestations, virilization as a result of androgen production by the tumor is rare. Here we present an extremely rare case of a sclerosing stromal ovarian tumor in a 14-year-old patient with bilateral presentation and with clinical and biochemical evidence of hyperandrogenemia.

## 1. Introduction

Sclerosing stromal tumor (SST) of the ovary is a rare benign tumor classified as a sex cord stromal tumor [1]. First described by Chalvardjian and Scully in 1973 [2], fewer than 100 cases have since been reported in the literature. The majority of cases occur in female patients under the age of 30 at mean age of 28 years [3]. SSTs are typically unilateral with bilateral presentation reported in only 2 cases [4]. SST usually presents with pelvic or abdominal pain and tenderness, a mass, and/or abnormal menses [5]. While hormonal activity has been reported predominantly in postmenarchal females with SST [6], only 5 cases presenting with virilization have been described [7]. In premenarchal girls, 7 cases of SST have been reported, with an age range from 7 months to 12 years. Of these, 3 demonstrated hormonal activity, with the 7-month-old female presenting with vaginal bleeding due to hyperestrogenism and the 11-year-old and 9-year-old females with virilization.

We present the third reported case of bilateral SST of the ovaries in a 14-year-old female and describe the ultrasound and MRI findings.

## 2. Case Report

A 14-year-old girl was referred for evaluation of primary amenorrhea and absence of breast development. Pubic and axillary hair development with body odor was described after age of 12 years. Physical examination revealed a lean, nondysmorphic female without acne or hirsutism, with Tanner IV pubic hair without clitoromegaly, and Tanner I breast development. Abdominal examination was benign.

Baseline laboratory testing demonstrated beta hCG of <1 mIU/mL (0–5), alpha fetoprotein of 2.9 ng/mL (0–8.3), and carcinoembryonic antigen of 1.3 ng/mL (0–4.7) (LabCorp, Raritan, NJ, USA). Additional testing showed luteinizing hormone of 2.0 mIU/mL, follicle stimulating hormone of 4.1 mIU/mL, and estradiol of 18 pg/mL; DHEAS was 420 mcg/dL, with 17-hydroxyprogesterone of 365 ng/dL and testosterone of 135 ng/dL (Esoterix Endocrinology, Calabasas, CA, USA). Chromosomal analysis revealed normal 46,XX karyotype (LabCorp, Raritan, NJ, USA).

**Figure 1:**
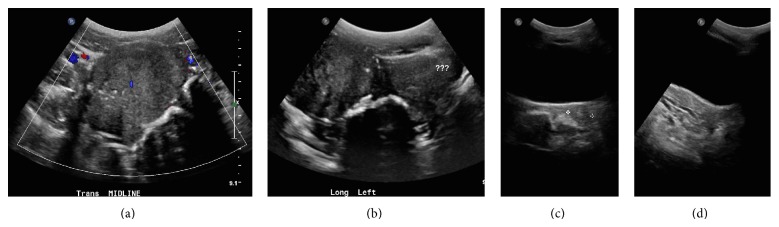
Transabdominal ultrasound imaging. (a), (b) Transverse and longitudinal images of large bilateral solid pelvic masses. Normal ovaries were not identified. (c), (d) Transverse and longitudinal images of a normal prepubertal uterus.

**Figure 2:**
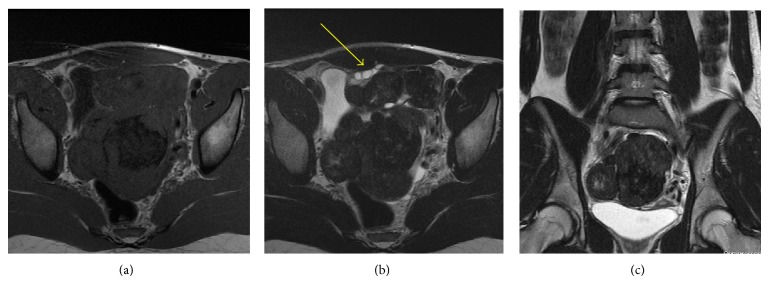
MRI imaging. (a) Axial T1-W image through the pelvis shows 1 contiguous or multiple soft tissue pelvic masses demonstrating isointense T1 signal when compared with normal muscle and a hypointense central component that represents the fibrous portion of the mass. (b) Axial T2-W image demonstrates multiple normal ovarian follicles within the anterior portion of the mass (*yellow arrow*), localizing the pelvic mass to the ovary. (c) Coronal T2-W image demonstrates heterogeneous solid masses predominately intermediate to low intensity T2 signal.

**Figure 3:**
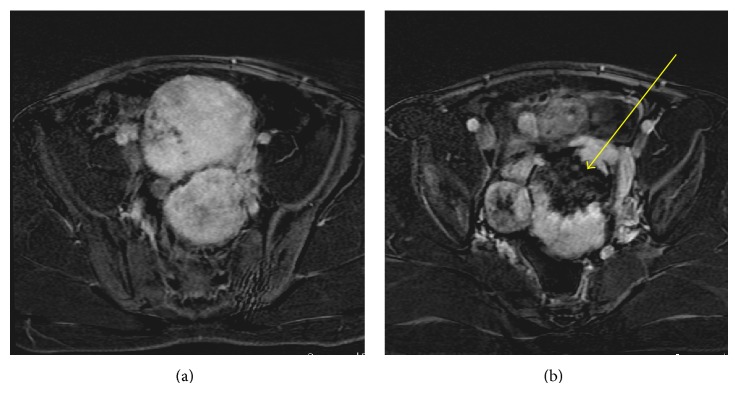
MRI imaging. (a), (b) Axial postgadolinium images demonstrate areas of significant enhancement within the pelvic masses with a central area of nonenhancement (*yellow arrow*) corresponding to the area of T1 hypointensity, which represents the fibrous portion of the tumor.

Upon presentation of initial imaging, transabdominal ultrasound images demonstrated large bilateral solid pelvic masses (Figures 1(a) and 1(b)). A small normal prepubertal uterus was imaged separate from the pelvic masses and normal ovaries were not clearly identified (Figures 1(c) and 1(d)). A follow-up gadolinium enhanced MRI of the abdomen/pelvis showed multiple soft tissue pelvic masses which are isointense to normal muscle on T1-weighted imaging with a central area of T1 hypointensity, intermediate to low signal intensity on T2-weighted imaging, and avid homogeneous predominantly peripheral enhancement on postgadolinium images (Figures 2(a), 2(c) and 3(a)). The central area of one of the masses which demonstrated isointense T1 signal did not enhance and probably represents a fibrous and acellular portion of the tumor (Figure 3(b)). Normal ovarian follicles were seen within the right ovary (Figure 2(b)). A prepubertal uterus was identified separate from the pelvic masses.

Intraoperative findings at laparotomy revealed abnormal appearance of both ovaries. The left ovary, which measured about 12 cm in greatest diameter, was replaced by an irregular, hard, calcified-appearing mass. The right ovary measuring 5 cm also appeared to be replaced by similar irregular, hard, calcified-appearing tissue. A left salpingo-oophorectomy was performed because the left ovary could not be salvaged. Dissection of the right ovary was performed with attempted removal of the abnormal tissue. The remaining right ovary was left in situ. A normal appearing, prepubertal sized uterus was noted. There was no palpable lymphadenopathy while the peritoneal surfaces were clear.

On gross examination, the left ovarian mass measured 11 × 9 × 8 cm and weighed 352 grams. Serial sectioning perpendicular to the longitudinal axis revealed multiple solid, adjacent nodules with focal calcification. These nodules were well circumscribed and encapsulated with white-tan, whorled cut surfaces with the largest nodule measuring 7 cm in greatest dimension. Normal ovarian tissue could not be identified. The right ovarian specimen weighed 50 g and consisted of firm, lobulated tissue measuring 8 × 4 × 4 cm. Serial sectioning perpendicular to the longitudinal axis revealed a large calcified central portion with the remainder of the specimen composed of 2 adjacent tan-white, homogeneous, whorled, solid nodules measuring 2 and 6 cm in greatest dimension.

**Figure 4:**
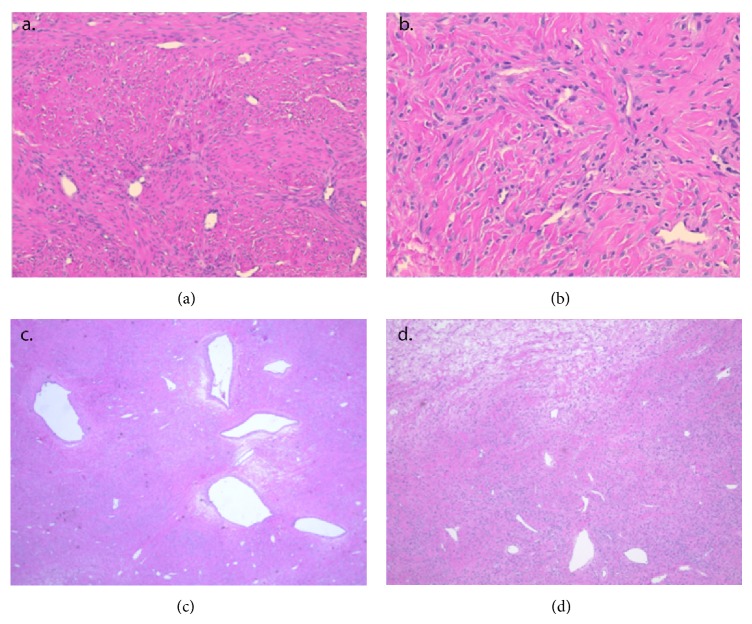
Hematoxylin and eosin stained slides. (a) Right ovarian mass with vague nodular outlines of pseudolobular architecture with variable hypercellular and hypocellular areas, 100x. (b) Right ovarian mass with marked vascularity composed of thin-walled and hemangiopericytoma-like vessels and interlobular fibrosis, 200x. (c) Left ovary with prominent ectatic vessels, 20x. (d) Left ovary with focal stromal edema (top left) and multiple ectatic and hemangiopericytomatous vessels, 40x.

Microscopic examination of both specimens revealed a pseudolobular architecture juxtaposed with biphasic hypercellular areas composed of bland collagen-producing spindle cells and hypocellular areas with focally edematous and fibrous stroma. Rare clusters of plump thecomatous cells with round nuclei and vacuolated eosinophilic cytoplasm were also noted interspersed throughout the stroma. Numerous, prominent thin-walled, and hemangiopericytoma-like (staghorn) vessels were also present within these bilateral tumors and in addition to rare ectatic (dilated) vessels in the left ovary. Although immunohistochemical stains were not performed, these morphological features along with the clinical history are most compatible with sclerosing stromal tumors of the bilateral ovaries (Figure 4).

Following an uncomplicated postsurgical course, follow-up 2 months after surgery revealed Tanner IV breast development, followed by menarche 1 month later. This has subsequently been followed by regular menses. Blood testing 3 months after surgery also revealed normal androgen and precursor levels.

## 3. Discussion 

Sex cord stromal tumors of the ovaries arise from embryonic sex cords or mesenchyme. The majority of ovarian sex cord stromal tumors are fibromas/thecomas (50%), granulosa cell tumors (10–20%), and Sertoli-Leydig tumors (5%).

Review of the literature (PubMed) reveals numerous case reports describing sonographic and MR findings of ovarian SSTs. Sonographic features include a multilocular cystic mass of heterogeneous or hypoechoic echogenicity with irregular thick septae. Early and strong peripheral enhancement is a key characteristic feature in distinguishing between SSTs and other types of sex cord stromal tumors. MR is the best modality for the imaging of pelvic tumors with a standard protocol that should include T2-weighted sequences and dynamic contrast-enhanced T1-weighted images with fat saturation in multiple imaging planes. T1-weighted images typically show intermediate signal in the outer part of the lesion and low signal intensity centrally. On T2-weighted images, there is intermediate to low signal intensity peripherally with interspersed areas of high signal intensity septae centrally within the mass. The peripheral band-like low signal intensity may reflect compressed ovarian tissue, which is a distinguishing feature between SSTs and fibromas/thecomas. The high signal intensity areas on T2-weighted images correlate with poorly cellular tissue (fibrous portion of the tumor). Early avid peripheral enhancement with centripetal progression and general lack of enhancement within the central area are usually seen on postcontrast images [5]. Our patient is unusual because the T1- and T2-weighted images show decreased signal abnormality within the central portion of the mass rather than interspersed areas of high signal intensity.

Histologically, SSTs of the ovary are characterized by distinctive features involving pseudo lobulated cellular and acellular zones with the cellular areas usually containing numerous branched vessels. Microscopic examination of this patient's tumors demonstrated typical findings for SST.

In conclusion, ovarian SSTs are very rare, benign, sex cord stromal tumors that typically present with a mean age of 28 years and usually are unilateral. Here we report an extremely rare case of an ovarian SST with bilateral presentation and with clinical and biochemical hyperandrogenemia, of which there are only 2 previously reported cases. Radiologically MR is the best modality for imaging of ovarian tumors with classic findings on T2-weighted images including intermediate to low signal intensity peripherally with interspersed areas of high signal intensity septae within the mass. Our case of SST is atypical as both ovarian masses demonstrate decreased T1 and T2 signal abnormality centrally with the mass.
